# Intraoperative Contrast Enhanced Ultrasound Evaluates the Grade of Glioma

**DOI:** 10.1155/2016/2643862

**Published:** 2016-03-16

**Authors:** Ling-Gang Cheng, Wen He, Hong-Xia Zhang, Qian Song, Bin Ning, Hui-Zhan Li, Yan He, Song Lin

**Affiliations:** ^1^Department of Ultrasound, Beijing Tiantan Hospital, Capital Medical University, 6 Tiantan Xili, Dongcheng District, Beijing 100050, China; ^2^Department of Neurosurgery, Beijing Tiantan Hospital, Capital Medical University, 6 Tiantan Xili, Dongcheng District, Beijing 100050, China

## Abstract

*Objective*. The aim of our study was to investigate the value of intraoperative contrast enhanced ultrasound (CEUS) for evaluating the grade of glioma and the correlation between microvessel density (MVD) and vascular endothelial growth factor (VEGF).* Methods*. We performed intraoperative conventional ultrasound (CUS) and CEUS on 88 patients with gliomas. All of the patients have undergone surgery and obtained the results of pathology. All patients have undergone intraoperative CUS and CEUS to compare the characteristics of different grade gliomas and the results of CUS and CEUS were compared with pathological results. *Results*. The time to start (TTS) and time to peak (TTP) of low grade glioma (LGG) were similar to those of edema and normal brain surrounding glioma. The enhanced extent of LGG was higher than that of the normal brain and edema. The TTS and TTP of high grade glioma were earlier than those of the edema and normal brain surrounding glioma. The enhancement of HGG was higher than that of LGG. The absolute peak intensity (API) was correlated with MVD and VEGF.* Conclusion*. Intraoperative CEUS could help in determining boundary of peritumoral brain edema of glioma. Intraoperative CEUS parameters in cerebral gliomas could indirectly reflect the information of MVD and VEGF.

## 1. Introduction

Glioma is the most common primary neuroepithelial tumors, which accounts for 50% of intracranial tumors [1]. The grade of glioma is closely related to the prognosis. The growth, invasion, and metastasis of tumor depended on angiogenesis, and the degree of tumor angiogenesis is closely related to malignancy and prognosis of tumor. CT can accurately determine the location of the lesion and clearly show the glioma calcification, but the accuracy is not good. MRI could diagnose the glioma more accurately than CT preoperatively. The image of MRI could detect small tumors which could not be displayed by CT. The assessment of gliomas by MRI was more accurate than CT, but it has limited application in surgery [2–4]. However it is difficult to distinguish the grade of glioma by CT or MRI preoperatively [5].

Glioma is one of the malignant tumors which are rich in angiogenesis. Invasive growth is one of the most important biological behaviors of the malignant tumor. Angiogenesis was significantly correlated with invasion and growth of the glioma. Microvessel density (MVD) is a quantitative criterion for reflecting the situation of tumor angiogenesis, and it can reflect the proliferation of tumor cells, angiogenesis, and the degree of malignancy objectively. Angiogenesis of tumor is a process regulated by gene and a variety of growth factors, in which the vascular endothelial growth factor (VEGF) is the most important blood vessel growth stimulating factor. However, MVD and VEGF of tumors are limited in clinical applications due to the invasive and poor reproducibility [6–9].

CEUS is the technique which can significantly improve the resolution, sensitivity, and specificity of ultrasound diagnostic. CEUS has become an important method of diagnosis, and it has been widely applied in the diagnosis of liver, kidney, and other organs but rarely used in the field of brain surgery. It could reflect and observe the blood perfusion of normal tissue and lesions [10, 11]. The residence time of contrast agents in intracranial benign and malignant tumor is different. It will certainly be helpful for the judgment of benign and malignant tumors according to the contrast agent development time-intensity curve [12–14].

Kanno et al. [15] did intraoperative CEUS in 37 patients with brain tumors; the results of vessels within the tumor had a good correlation between CEUS and digital subtraction angiography (DSA), but now the studies of distinguishing gliomas grading by CEUS were few. The purposes of this study were to explore the value of intraoperative CEUS in the evaluation of peritumoral edema and tumor grading, while evaluating relationship between contrast enhanced ultrasound parameters and MVD or VEGF in different pathologic grades of cerebral gliomas.

## 2. Materials and Methods

### 2.1. Patients

88 patients were selected from April 2009 to December 2014 in the Beijing Tiantan Hospital neurosurgery. They were all diagnosed as having supratentorial gliomas by CT and/or MRI. They were 56 males and 32 females and aged from 18 to 69 years, with a mean age of 45.2  ±  12.8 years. Clinical manifestations were headache, limb weakness, limb twitching, blurred vision, aphasia, and so on. This study was approved by our local ethics committee, and written informed consent was obtained from each patient before the CEUS examination and biopsy procedures. The necessity and function of the ultrasonic imaging examination and the possible side effects were explained to patients or patients' families. “CEUS informed consents” were signed by the patients or their immediate family members.

### 2.2. Preoperative CT and/or MRI

All of the tumors whose diameters were from 2.1 to 5.4 cm were single. There were 33 cases of frontal gliomas, 19 cases of temporal gliomas, 2 cases of parietal gliomas, 4 cases of occipital gliomas, 15 cases of frontotemporal gliomas, 12 cases of temporal parietal gliomas, 1 case of thalamus glioma, and 2 cases of frontal and parietal gliomas.

### 2.3. Inclusion and Exclusion Criteria

#### 2.3.1. Inclusion Criteria

The inclusion criteria are as follows: (1) all patients were suspected as glioma with preoperative CT and MRI; (2) the selected gliomas were those which had less clear border and supratentorial gliomas, especially those that had edema; (3) the diameter of gliomas is <5.5 cm; (4) all of the patients voluntarily join this study and are older than 18 years.

#### 2.3.2. Exclusion Criteria

The exclusion criteria are as follows: (1) the patients who refused to participate in this study; (2) the patients who were allergic for contrast agent composition; (3) the patients that were not suitable for participation in the study because of severe heart and lung disease; (4) the quality of conventional ultrasound image that was not satisfactory; (5) the patients who had received other treatment before surgery, such as radiotherapy and chemotherapy.

### 2.4. Instruments and Reagents

The ultrasound scanner is *α*-10 (Aloka, Japan), equipped with UST-9133 (transducer surface 3.0 cm × 1.0 cm), and the frequency of probes is 6–8 MHz, and its maximal depth is 18 cm. CEUS analysis software was installed into the ultrasound scanner. Ultrasound output mechanical index was 0.10–0.12.

Ultrasound contrast agents were SonoVue produced by Bracco Company, Italy. The agents were microbubbles of the phospholipids microencapsulated sulfur hexafluoride (SF_6_). The average diameter of microbubbles was 2.5 *μ*m and their pH values ranged from 4.5 to 7.5. 5 mL of 0.9% sodium chloride was injected into 59 mg of SonoVue before contrast and then thoroughly shaken. After 5 mL contrast agent was injected from femoral vein, 10 mL saline was injected immediately.

### 2.5. Inspection Method

#### 2.5.1. Surgery

Under general anesthesia, the neurosurgeon shaved the hair and cleaned the skin in the surgical area. The neurosurgeon then made an incision through the scalp at the location of the glioma according to preoperative head CT/MRI. Brain surgery was performed through the bone flap after opening the skull.

The intraoperative ultrasound probe was placed into a sterile transducer cover (Surgical Sterile Protective Ship-Cover, 3L Medical Products Group Co., Ltd., Jiangxi, China) and was then inserted into the bone flap to observe the glioma after opening the calvarium and tenting the dura. The pressure on the brain was minimized as much as possible.

The doctor detects lesion directly on the brain surface that uses saline as a coupling agent after cutting the cerebral dura mater; multislice examination of the lesions was rowed within the range of bone window. We observed the location, relationship boundaries, shape, internal echo, peripheral edema brain tissue and normal brain tissue, tumor size, depth from the brain surface, blood flow characteristics, and the necrosis of glioma by using intraoperative CUS.

Ultrasound scanner setting was switched to CEUS after the best section of lesion displayed. The target was in the center of the screen and regulated the depth of scanner focus, and so forth. Five milliliters of contrast agent was bolus injected via the femoral vein and then 10 mL of saline was injected for washing. The timer on ultrasound scanner was started at the same time of contrast agent injection. The characteristics of enhancement in glioma and surrounding brain tissue during the administration of ultrasound contrast agents were observed in real time for 2 min. The characteristics of the glioma on real time CEUS were observed. The real time images of CEUS were stored on cine loops and static images.

Remove cotton sheets, tissue debris, and blood clot in residual cavity after surgery. The residual cavity was filled with saline after repeated washing and observed whether the glioma was removed completely or not.

Intraoperative CUS and CEUS were done by the same doctor, and the injection of contrast agent was also by the same nurse.

#### 2.5.2. Postoperative Image Handling

All raw data were stored in the instrument's hard drive and then analyzed by the time-intensity curve (TIC) software equipped in the machine. A plurality of regions of interest was analyzed and compared, in order to get the time to start (TTS), time to peak (TTP), the absolute peak intensity (API) of glioma, peritumoral edema, and surrounding normal brain tissue.

### 2.6. Image Analysis

Five values were obtained about every glioma, peritumoral edema, and normal brain tissue for TIC analysis. The regions of interest (ROI) were circularity whose diameter was 0.9 cm. ROI were selected avoiding the area of necrosis of lesion. The following parameters were obtained: the time to start (TTS), which means the time of interesting regions starts to be enhanced; the time to peak (TTP), which means the time of interest regions is enhanced to the peak; the absolute peak intensity (API), which is equal to peak intensity-baseline intensity. The average values of all the 5 were obtained as the TTS, TTP, and API of the ROI. The images were analyzed by two experienced sonographers and they reached an agreement about the results.

### 2.7. Immunohistochemical Detection of MVD and VEGF

#### 2.7.1. Reagents of Pathology

They are biotinylated goat anti-human VEGF polyclonal antibody (CYB165004), mouse anti-human CD34 monoclonal antibody (SPM123), SABC immunohistochemistry kit (SA1020), and DAB substrate kit (PW017). Pathological images were received by image analysis system and radiography.

#### 2.7.2. Quantification of MVD

There were two ways of treatment for postoperative specimens: (1) conventional sections after being fixed with formalin, and then embedded with paraffin, and HE staining and (2) 4 *μ*m thick paraffin sections that were stained by anti-CD34 monoclonal antibody peroxidase labeled avidin-linked enzyme. The result and statistics were got by two pathologists who had extensive clinical experience in double blind method.

The standards of MVD count were referring to count technique proposed by Yu et al. [16]. Microvessels which were counted contained single endothelial cells which were dyed brown single endothelial cells, endothelial cell clusters into the lumen, and even larger vessels, as long as it was separated from the neighboring capillaries, tumor cells, or other connective tissues. The number would be counted as two microvessels if the “head” and “tail” of the same vessel are displayed in the same plane; the vessel would be counted as a blood vessel if there were less than 8 caught red blood cells in the luminal diameter; if there were >8 red blood cells or smooth muscle wall, the vessels were not counted. First “hot spots” which were glioma cell infiltration and areas that contain most microvessels were selected at low magnification. “Hot spots” were generally common in the edges of glioma. The MVD would be counted under high magnification vision after finding “hot spots”; every sample would count number of microvessels in five horizons (counting units: bar/HPF), the average of which was as the MVD of the glioma.

#### 2.7.3. Expression of VEGF

Postoperative specimens were fixed in 10% formalin and embedded by paraffin and then biotinylated goat anti-human VEGF polyclonal antibody as an antibody. The positive staining of VEGF was that there was granular brown substance in the cytoplasm or nucleus. Each slide was counted five high power fields randomly. Each field counted 200 cells. The average positive rate = the number of positive cells/ the number of counted cells. The result was the average of five visions. Positive grading criteria are as follows: positive cell rate between 0% and 10% was (−); 11% to 40% was (+); 41% to 75% was (+); higher than 76% was (+++).

### 2.8. Statistical Analysis

SPSS19.0 statistical software was used to analyze the results. Normal measurement data were representing mean ± standard deviation. Measurement data were compared by *t*-test or analysis of variance. Count data were compared by *χ* ^2^ test or rank sum test. *P* < 0.05 was considered statistically significant.

## 3. Results

### 3.1. The Basic Condition of the Patients

All patients obtained a clear image of CEUS, and all patients were well tolerated about CEUS. There were no adverse reactions such as dizziness, headache, abdominal pain, feeling strange, joint and muscle pain, and weakness during and after the examination.

All of 88 patients were glioma confirmed by pathology. Low grade gliomas (LGG) included levels I and II, and high grade glioma (HGG) mainly included levels III and IV referring to the WHO classification of glioma in 2000. There were 38 cases of low grade gliomas and 50 cases of high grade gliomas (Table 1), 56 males and 32 females, aged from 20 to 69 years, with a mean age of 47.9  ±  11.4. The area of edema appeared in 7 cases in low grade glioma and 22 cases in high grade glioma.

### 3.2. The Intraoperative CUS and CEUS Performance of Gliomas

The CUS showed that the glioma is hyperechoic, the boundary is not clear, and the shape is irregular. The echo of peripheral edema was lower than that of glioma but still higher than that of normal brain tissue. The boundaries between glioma and edema were unclear. The area of intralesional necrosis presented as hypoechoic, which was lower than the surrounding brain parenchyma. CDFI showed that there was little blood flow in the glioma.

**Figure 1:**
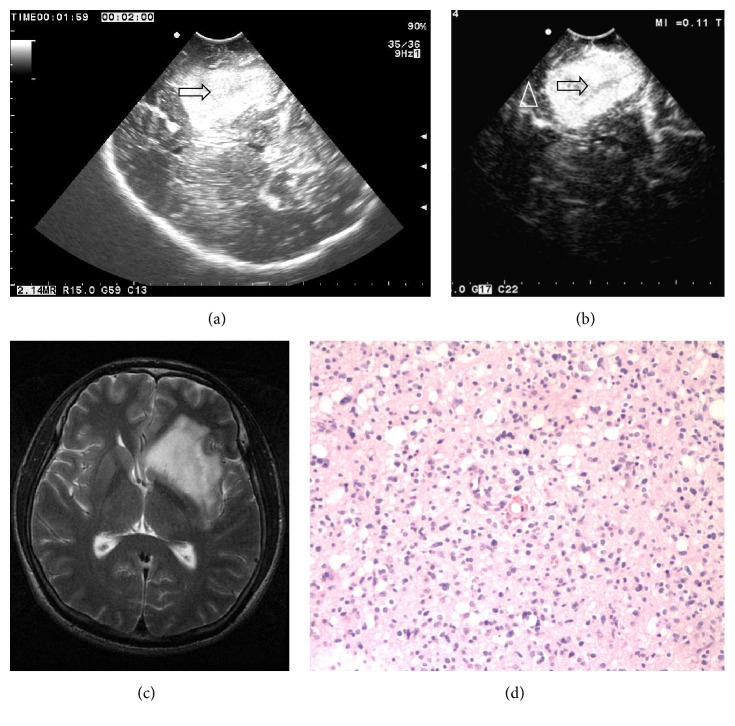
(a) Ultrasound image shows that the low grade glioma (II level) is partially hyperechoic, the boundary is not clear, internal echo is nonuniform, and edema is not obvious (arrow showed tumor). (b) CEUS shows that the echo of the glioma is enhanced significantly, the boundary is clear, and normal brain tissue is enhanced lower than the glioma (arrows show tumor; triangle shows normal brain tissue). (c) MRI T2W image shows that the glioma is hyperintense, irregular, and the boundary is not clear. (d) The pathological image of the same patient shows that the number of capillary vessels is less (HE ×100).

**Figure 2:**
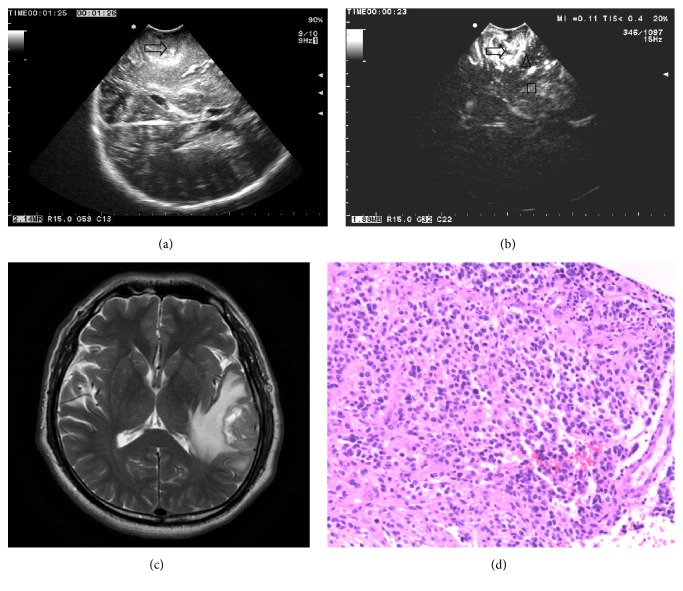
(a) Ultrasound image of high grade glioma shows that the tumor is hyperechoic, and the boundary is not clear and heterogeneous internal echo. The edema is obvious (arrow tumor). (b) CEUS shows that the echo of the glioma is enhanced significantly, brain edema is enhanced lower than the glioma, and brain tissue is enhanced lower than the glioma and edema (arrow showed the glioma, brain edema triangle shown, and the box showed normal brain tissue). (c) MRI T2W shows that the high grade glioma is mixed-signal, and there is edema around it. (d) The pathological image of the same patient shows that the microvessel is abundant (HE ×100).

88 patients with glioma injected contrast agent via femoral venous. TTS of LGG was from 6 s to 18 s, TTP of which was from 12 s to 28 s; TTS of HGG was from 4 s to 14 s, and TTP of HGG was from 10 s to 24 s. CEUS features of LGG are as follows: the TTS and TTP of glioma and edema and normal brain surrounding glioma were similar. The enhancement of glioma was uniform or nonuniform and higher than the normal brain and edema. Peritumoral edema showed equal enhancement approximately. The surrounding normal brain tissue showed equal enhancement (Figure 1). CEUS features of HGG are as follows: the TTS and TTP of glioma were earlier than the edema and normal brain surrounding glioma. The enhancement of glioma was uniform or nonuniform and higher. Peritumoral brain edema was highly enhanced. The boundary of glioma was clear with peritumoral edema brain tissue and surrounding normal brain tissue (Figure 2).

### 3.3. Time-Intensity Curve of Intraoperative CEUS (TIC)

**Figure 3:**
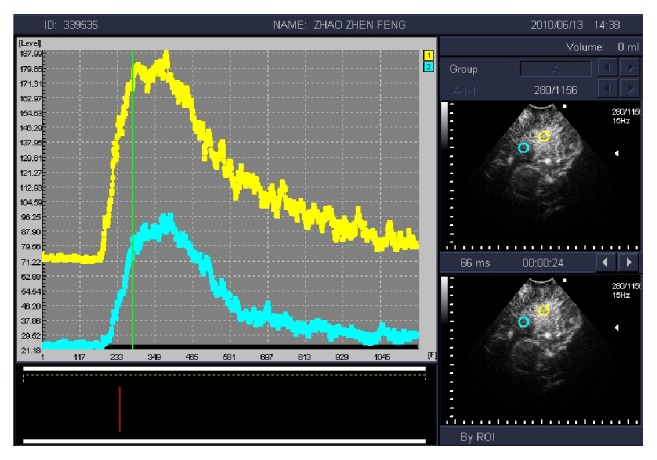
Time-intensity curve (TIC) of low grade glioma (yellow) shows that contrast peak intensity of glioma is significantly higher than the surrounding normal brain tissue (blue).

**Figure 4:**
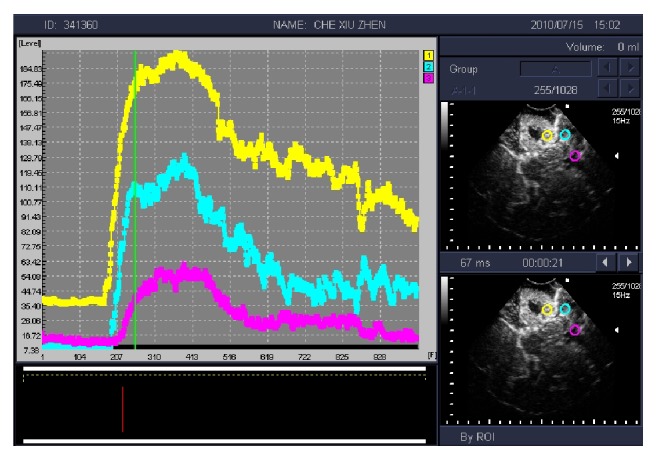
Time-intensity curve (TIC) of high grade glioma shows that tumor tissue (yellow) is significantly higher than the peak intensity of contrast peritumoral brain edema (blue) and the surrounding normal brain tissue (pink).

The API of LGG was higher than the surrounding normal brain tissue and peritumoral brain edema, and it was statistically significant between the two groups (*P* < 0.05). The difference of TTS and TTP between the glioma and the surrounding normal brain tissue was not statistically significant (*P* > 0.05) (Table 1, Figure 3). The API of HGG was higher than the surrounding normal brain tissue and peritumoral brain edema significantly. It was statistically significant between the two groups (*P* < 0.05). The difference of TTS and TTP between the glioma and the surrounding normal brain tissue was statistically significant (*P* < 0.05) (Table 1, Figure 4).

There was cerebral edema appearing in 7 cases of LGG. The difference of TTS and TTP among glioma, peritumoral edema, and the surrounding normal brain tissue was not statistically significant (*P* > 0.05) (Table 2). API of edema had statistically significant difference compared with glioma (*P* < 0.05), and it had no statistically significant difference compared with the surrounding normal brain tissue (*P* > 0.05). There were 22 patients in HGG who could find cerebral edema around the glioma. The difference of TTS and TTP among glioma, peritumoral edema, and the surrounding normal brain tissue was not statistically significant (*P* > 0.05) (Table 2). API of edema had statistically significant difference compared with glioma and the surrounding normal brain tissue (*P* < 0.05).

There was no statistically significant difference about TTS in glioma and the surrounding normal brain tissue between LGG and HGG. The difference of TTP and API was statistically significant (*P* < 0.05) (Table 3). The TTS, TTP, and API of edema had no statistically significant difference between LGG and HGG (Table 3).

### 3.4. Relationship between Glioma of CEUS and MVD

**Figure 5:**
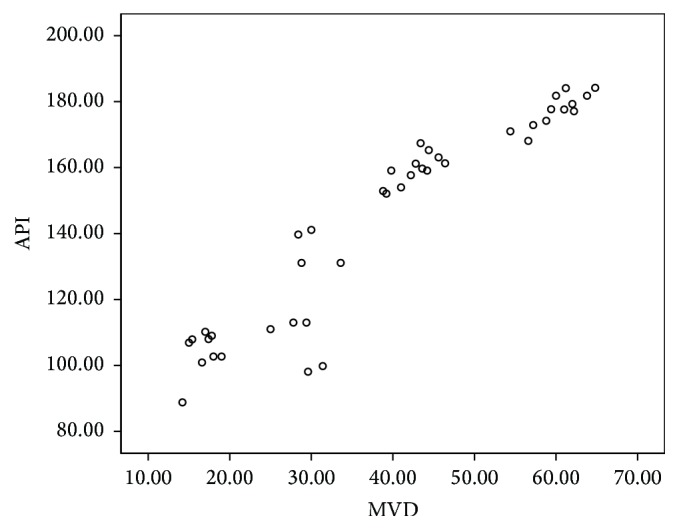
Correlation of absolute peak intensity (API) and microvessel density (MVD) about glioma tumor.

All the microvessel endothelial cells of 38 cases showed that anti-CD34 antibody staining was positive. MVD of 42 cases is from 14.20 to 64.80. MVD of high grade (III, IV) glioma was significantly higher than the low ones. The difference between the two groups was statistically significant (*P* < 0.05) (Table 4). API of glioma showed a positive correlation with MVD (*r* = 0.899, *P* = 0.000; Figure 5).

### 3.5. Relations between Different Pathological Grade Gliomas with VEGF

The expression of VEGF about normal brain tissue was negative. The expression of VEGF staining positive was located in the cytoplasm of glioma tumor cells and endothelial cells. VEGF of HGG was higher than the LGG; the difference between groups was statistically significant (*P* < 0.05) (Table 4).

## 4. Discussion

Glioma is the most common malignant brain tumor, whose basic treatment is surgery combined with radiotherapy and chemotherapy. Whether the glioma is removed completely or not is directly related to the prognosis of patients. Radical tumor surgery is important for improving the clinical outcome and keeping neurological function at the same time [17, 18]. At present, many advanced imaging techniques have been used in the field of neurosurgery surgery, such as nerve navigation, intraoperative CT, and MRI. These methods are not yet widely used in surgery limited by various conditions. Intraoperative CUS not only has the advantages of being cheap, convenience, and repeatability, but also can get real time imaging fully synchronized with surgical procedures [19]. However there was lack of clear boundary and capsule around the glioma due to the biological characteristics of the invasive growth of glioma. It is difficult for intraoperative CUS to distinguish the boundary of glioma, especially for distinguishing the boundary of residual glioma with peritumoral edema [20, 21]. It is difficult to reduce the residual tumors and increase tumor total resection rate just by using intraoperative CUS. In this study, CEUS was applied in glioma surgery. It not only improves the ability of intraoperative ultrasound to identify the glioma, residual glioma, and peritumoral edema, but also can judge the tumor pathological nature preliminary.

Because of the biological characteristics of the invasive growth of glioma, we found that it has varying degrees of edema around some tumor tissue. It is difficult to distinguish tumor boundary between peritumoral edema and normal brain tissue by intraoperative CUS. It may lead to unnecessary brain damage, if the edema brain tissue was removed mistakenly as tumor. At present, the formation mechanism of peritumoral edema is not yet clear. Some scholars [22, 23] noted that the most generation of edema is of vascular origin. Some scholars [24–26] believe that toxic tissue edema due to glioma cells produces abnormal capillaries leaking. Different pathological grade gliomas had different ultrasound contrast images. The results of this study show that the number of peritumoral edema cases of HGG was significantly more than LGG. 22 cases of HGG have peritumoral edema, and only 7 cases of LGG have peritumoral edema. We found that the TTS and TTP of LGG, edema, and normal brain surrounding glioma were similar in CEUS. The enhancement of glioma was uniform or nonuniform, and it is higher than the normal brain and edema. Peritumoral edema showed equal enhancement approximately. The surrounding normal brain tissue showed equal enhancement. The TTS and TTP of HGG were earlier than the edema and normal brain surrounding glioma. The enhancement of glioma was higher. Peritumoral brain edema was highly enhanced. The boundaries of glioma were clear with peritumoral edema brain tissue and surrounding normal brain tissue. Thus we can distinguish tumor with peritumoral edema and the brain tissue by intraoperative CEUS of glioma, which could improve the diagnosis of the glioma and brain tissue edema compared to IOUS. The higher the malignancy degree of gliomas, the more abundant the blood vessels of gliomas [27–29].

Angiogenesis was significantly increased with the increase of the degree of malignancy, which could result in microvessel increase and the structure of abnormal new blood vessels. MVD was the gold standard to evaluate tumor angiogenesis, which can reflect tumor cell proliferation, angiogenesis, and the degree of malignancy, and so on objectively. It is an important indicator for evaluating brain tumor biological behavior and prognosis by the clinical and molecular pathology. CD34 is widely distributed in the tumor vascular endothelial cells in gliomas. Tiny tumor blood vessels can be identified by CD34 staining [25, 30]. However, MVD is limited in the clinical application due to the invasive and poor reproducibility. TIC of CEUS had rich quantitative information. It can reflect tumor blood perfusion, and it becomes the latest inspection methods of evaluation of tumor blood vessels [31, 32]. Tumor blood supply is more abundant; the amount of blood flow increased with the increase of the number of tumor angiogeneses. The degree of enhancement of tumor was more obvious. This study found that API was positively correlated with MVD. Thus, quantitative parameters of CEUS about glioma may indirectly reflect the hemodynamic characteristics and MVD of glioma, which can evaluate glioma angiogenesis reliably and noninvasively, and determine pathological level and provide valuable information for clinical treatment. Angiogenesis is the process regulated by gene and a variety of growth factors. Malignant glioma cells could secrete large amounts of VEGF, stimulate endothelial cell proliferation and migration, and then generate new tumor blood vessels. Nakada et al. [26] found that angiogenesis can be inhibited by inhibiting VEGF and matrix metalloproteinase activity. This study shows that VEGF expression in HGG group was significantly higher than the LGG group, suggesting that VEGF are closely related to the invasiveness and malignancy of glioma.

## 5. Study Limitations

(1) Only a small part of typical glioma was examined by CEUS intraoperative CEUS. This paper summarizes the CEUS characteristics of gliomas and is not comprehensive enough, and it needs to be supplemented. (2) It is only a preliminary judgment of pathological grade about glioma by CEUS, and we do not do the pathological grade. We hope that it could be further studied and solved by expanding the sample size and histological type.

## 6. Conclusion

Intraoperative CEUS can show the boundary of glioma clearly. TTS, TTP, and API of glioma, peritumoral brain edema, and normal brain tissue were quantitatively analyzed. API was positively correlated with MVD and VEGF.

## Figures and Tables

**Table 1:** The comparison of CEUS about different grade gliomas.

Sampling sites	LGG			HGG		
	TTS	TTP	API	TTS	TTP	API
Cancer	11.11 ± 3.36	19.56 ± 4.27	147.48 ± 46.29	10.26 ± 2.82	17.34 ± 3.68	171.22 ± 29.34
Peritumoral normal tissue	11.21 ± 3.59	19.84 ± 4.68	81.88 ± 29.49	11.72 ± 2.76	19.36 ± 3.66	71.74 ± 24.23
*t* value	0.56	1.70	11.65	5.99	6.65	19.65
*P* value	0.58	0.09	0.000	0.000	0.000	0.000

**Table 2:** The comparison of brain edema about different grade gliomas.

Sampling sites	LGG			HGG		
	TTS	TTP	API	TTS	TTP	API
Cancer	11.19 ± 2.44	19.29 ± 3.80	154.64 ± 31.71	9.99 ± 3.12	17.29 ± 3.77	177.12 ± 31.79
Peritumoral edema	12.19 ± 2.30	19.49 ± 3.87	111.37 ± 29.42	11.51 ± 2.62	19.55 ± 3.08	107.88 ± 19.13
Peritumoral normal tissue	11.45 ± 3.44	19.39 ± 3.73	81.92 ± 29.71	11.99 ± 2.66	19.41 ± 3.20	63.25 ± 21.30
*F* value	0.24	0.01	10.20	3.03	3.11	111.62
*P* value	0.78	0.99	0.001	0.06	0.051	0.000

**Table 3:** Comparison of different grade gliomas and edma by CEUS.

Grade	Gliomas			Edma		
	TTS	TTP	API	TTS	TTP	API
LGG	11.11 ± 3.36	19.56 ± 4.27	147.48 ± 46.29	12.19 ± 2.30	19.49 ± 3.87	111.37 ± 29.42
HGG	10.26 ± 2.82	17.34 ± 3.68	171.22 ± 29.34	11.51 ± 2.62	19.55 ± 3.08	107.88 ± 19.13
*t* value	1.29	2.61	2.93	0.61	0.04	0.37
*P* value	0.20	0.01	0.004	0.55	0.97	0.72

**Table 4:** The comparison of MVD and VEGF about different pathological grade glioma.

Grade	Number (*N*)	MVD	VEGF			
			−	+	++	+++
Low grade glioma	18	23.02 ± 6.78	7	7	3	1
High grade glioma	24	51.37 ± 9.34	0	3	9	12

*P* < 0.05; the difference of MVD and VEGF was statistically significant.
