# Corrigendum to “Intraoperative Contrast Enhanced Ultrasound Evaluates the Grade of Glioma”

**DOI:** 10.1155/2017/5782619

**Published:** 2017-07-18

**Authors:** Ling-Gang Cheng, Wen He, Hong-Xia Zhang, Qian Song, Bin Ning, Hui-Zhan Li, Yan He, Song Lin

**Affiliations:** ^1^Department of Ultrasound, Beijing Tiantan Hospital, Capital Medical University, 6 Tiantan Xili, Dongcheng District, Beijing 100050, China; ^2^Department of Neurosurgery, Beijing Tiantan Hospital, Capital Medical University, 6 Tiantan Xili, Dongcheng District, Beijing 100050, China

In the article titled “Intraoperative Contrast Enhanced Ultrasound Evaluates the Grade of Glioma” [1], there were errors in the Materials and Methods and Results sections, as mentioned in a Letter to the Editor by Mahalangikar et al. [2], which should be corrected as follows.

In Section  2.7.3 (Expression of VEGF), “41% to 75% was (+)” should be corrected to “41% to 75% was (+ +).”

The age ranges were incorrectly reported in Section  3.1 (The Basic Condition of the Patients) as “20 to 69 years with a mean age of 47.9 ± 11.4” and should be corrected to “18 to 69 years with a mean age of 45 ± 12.8 years.”
